# Bioinformatics analysis of multi-epitope peptide vaccines against Hepatitis C virus: a molecular docking study

**DOI:** 10.1186/s43141-023-00583-w

**Published:** 2023-11-14

**Authors:** Ashraf M. Muhammad, Ghada M. Salum, Mai Abd El Meguid, Basma E. Fotouh, Reham M. Dawood

**Affiliations:** 1 Applied Biotechnology Program, Faculty of Science, Ain Shams University, Cairo, 11566 Egypt; 2 Department of Microbial Biotechnology, Genetic Engineering Division, National Research Centre, Dokki, P.O. 12622, Giza, Egypt

**Keywords:** HCV, Humoral response, Cellular responses, CD4, CD8, Toxicity, Antigenicity, Docking scores, Vaccine development

## Abstract

**Background:**

Hepatitis C Virus (HCV) infection is one of the causal agents of liver disease burden. Six multiple antigenic peptides were synthesized including (P315, P412, and P517) plus (P1771, P2121, and P2941) to induce humoral and cellular responses, respectively against HCV infection. Aim: This paper aimed to employ computational tools to evaluate the efficacy of each peptide individually and to determine the most effective one for better vaccine development and/or immunotherapy.

**Methods:**

VaxiJen web and AllerTOP servers were used for antigenicity and allergenicity prediction, respectively. The ToxinPred web server was used to investigate the peptide toxicity. Each peptide was docked with its corresponding receptors.

**Results:**

No peptides were expected to be toxic. P315 and P2941 are predicted to have robust antigenic properties, lowest allergenicity, and minimal sOPEP energies. In turn, P315 (derived from gpE1) formed the highest hydrophobic bonds with the BCR and CD81 receptors that will elicit B cell function. P2941 (derived from NS5B) was shown to strongly bind to both CD4 and CD8 receptors that will elicit T cell function.

**Conclusion:**

P315 successfully bound to B cell (BCR and CD81) receptors. Also, P2941 is strongly bound to T cell (CD4 and CD8) receptors.

## Background

Hepatitis C Virus (HCV) infection was first known in 1989 by researchers who recently won the Nobel Prize [1–3]. HCV is one of the causal agents of liver disease burden including chronic hepatitis, cirrhosis ending with hepatocellular carcinoma [4–6]. World Health Organization recorded around more than seventy million infected patients worldwide with at least 400,000 cases of death annually [1]. HCV is a positive-strand RNA virus. Its enveloped genome counts around 96 kilobases. After viral cleavage, viral proteins including the capsid, two envelope proteins (E1 and E2), and seven non-structural proteins will be identified [7]. HCV infection treatment has successfully improved from poorly tolerated injectable therapy (pegylated interferon and ribavirin) to well-tolerated oral direct-acting antiviral therapy (DAAs) [8, 9]. The great virologic cure rate was attained through an improved understanding of the viral lifecycle and the recognition of its targets to be blocked by small molecules such as polymerase and protease inhibitors [10]. Vaccination would have a significant impact on efforts for eradicating HCV infection and aiding as a complementary strategy [11].

Several approaches are currently being used in the development of HCV vaccines such as recombinant proteins, peptide vaccines, DNA vaccines, virus-like particles (VLPs), and viral vectors expressing diverse antigens. It is interesting to note that VLPs are becoming desirable candidates for the development of HCV vaccines due to their ability to strongly stimulate cellular and humoral immune responses [12]. However, more research is needed to determine how to trigger immune responses that are highly protective and long-lasting. The NS3, NS4, NS5, and core proteins, which are targets of CD8 + T cells, are typically the focus of vaccines that induce T cell-mediated immunity. The pre-clinical studies that simply looked at T-cell responses were unsuccessful [13, 14].

A promising vaccine composed of NS proteins in chimpanzee adenovirus (ChAd3-NS) was tested in human volunteers and boosted with modified vaccinia Ankara virus (MVA-NS). Broadly HCV-specific memory CD4 + and CD8 + T cells were produced by this treatment [15]. The clinical trial of this vaccine, a phase 1/2 trial in PWIDs (ClinicalTrials.gov identifier NCT01436357), showed no protection in patients with chronic HCV infection.

The HCV glycoproteins E1, E2, or the E1E2 heterodimer, which are the primary targets of protective broad-spectrum neutralizing antibodies (bnAbs), are the basis for vaccines intended to elicit humoral immune responses. The most successful candidate for this strategy to date is a pure recombinant E1E2 (rE1E2) protein based on the HCV genotype 1a. After a homologous challenge, this vaccination provided protection in chimpanzees [16], while a heterologous challenge resulted in lower rates of persistence [17]. Humans were unharmed by the rE1E2 protein in an oil-in-water emulsion, and only three of 16 vaccinated people experienced bnAb response [18, 19]. Recently, Patra et al. (2023) examined the immunogenicity of the antigen mRNA-lipid nanoparticles (LNPs) expressing the soluble E1 and E2 in the mouse model [20]. They proved that the use of both E1 and sE2F442NYT mutant can induce broad protective humoral and cellular immunity indicating that the mRNA-LNP platform can provide a good opportunity to be used as an efficient candidate vaccine.

Peptide vaccines are short amino acid sequences that depict a particular epitope of an antigen and are designed to trigger an immune response to that antigen. Since viral proteins contain epitopes as their antigenic determinants, epitope-based peptide vaccines can elicit cellular and humoral responses without developing undesirable adverse reactions [21]. Peptide vaccines provide several advantages in comparison to traditional vaccines that include whole viral particles or large portions of viral proteins. The use of traditional vaccinations requires adding an extra antigenic load that mildly stimulates the immune system and results in allergic responses. Peptide vaccines, on the other hand, use small portions of the viral antigens, that are non-infectious on their own, to drive more targeted and effective immunogenic reactions and, as a result, prevent allergic ones [22].

The field of Bioinformatics provides a broad range of tools that make it easier and more efficient to produce peptide vaccines cheaply and rapidly. In the design of peptide vaccines, finding correct epitopes is a vital process that necessarily involves sequence analysis to assess the amino acids in the pathogenic proteins and identify the proper motif [23, 24]. Immunogenicity is a crucial component of vaccines that helps to stimulate a powerful immune response, despite, low immunogenicity is one of the drawbacks of peptide vaccines [25, 26]. Accordingly, employing computational models to predict and determine the immunogenicity of the proposed peptides is a fundamental step in vaccinology [22, 27, 28]. Safe vaccines must be non-allergic and non-toxic by nature. In order to predict the potential allergenicity and toxicity of the epitopes, several computational methods, including alignment-based and -free methods, were developed [26, 29]. Finally, molecular docking is a widely used effective technique to predict and evaluate the interaction between the peptide and their targets [30, 31]. Despite significant developments in recent years, there is no commonly established framework for vaccine design in silico.

Firstly, the peptides were assessed for antigenicity, allergenicity, and toxicity to get proper information regarding their immunogenicity. Then, stable tertiary structures of these peptides were modeled and validated to be docked with their targeted receptors. Finally, docking scores as well as the chemical bonds involved in the peptide-protein interactions were reported to validate their ability in inducing immune responses upon injection.

## Materials and methods

### Peptides characterization

All six peptides, summarized in Table 1, were assessed for antigenicity, allergenicity, and toxicity using their amino acid sequence as input. For antigenicity prediction, the VaxiJen web server was used to employ models for predicting protein antigenicity from protein datasets with an alignment-free algorithm [32, 33]. For the evaluation of allergenicity, the AllerTOP v.2.0 server that predicts the allergenicity from the amino acid hydrophobicity, molecular size, helix-forming propensity, the relative abundance of amino acids, and β-strand forming propensity, was employed [34]. For toxicity prediction, the ToxinPred web server was used to investigate the peptides’ toxicity by evaluating the toxicity for all potential variants of the input sequences using models based on machine learning techniques and quantitative matrices [35]. In addition, the prediction of the peptides’ hydrophobicity and half-life in blood was performed using the PlifePred web server utilizing 261 peptides with experimentally determined half-lives in the mammalian blood [36].

**Table 1:** Sequence identity of the selected peptide epitopes as compared with corresponding regions derived from genotypes 2a (JFH1) and a chimeric 2a/4a virus (ED43/JFH1)

Peptide name	Derived protein	Amino acid position	Peptide sequence
P315	E1	a.a. 315–326	GHRMAWDMMMNW
P412	E2	a.a. 412–423	QLINSNGSWHIN
P517	E3	a.a. 517–531	GTTDHVGVPTYDWGK
P1771	NS4B	a.a. 1771–1790	GIQYLAGLSTLPGNPAIASL
P2121	NS5A	a.a. 2121–2140	FFTEVDGIRLHRHAPKCKPL
P2941	NS5B	a.a. 2941–2960	CGIYLFNWAVKTKLKLTP

### 3D modeling and structure validation

The peptide sequences were modeled to a tertiary structure using the PEP-FOLD web server [37, 38]. The best 3D models are determined based on their sOPEP (Optimized Potential for Efficient structure Prediction) energy expressed as a sum of local, nonbonded, and hydrogen-bond terms (Equation) [37, 39].

$$E={E}_{local}+{E}_{nonbonded}+{E}_{H-bond}$$

### Molecular docking of the peptides with their corresponding receptors

The immune receptor structures were retrieved from RCSB PDB. The first three peptides (P315, P412, and P517) were docked against the B-cell receptor (BCR) (ID: 5DRX) and CD81 (ID: 3X0E), while the other three (P1771, P2121, and P2941) were docked with the T-cell receptors (CD4 and CD8 (IDs: 2NY1 and 3QZW)). The protein structures were cleaned by removing water molecules and unnecessary structures and optimized by energy minimization with SWISS-PDBViewer [40]. The docking simulations were performed with a hybrid method combining template-based modeling and ab initio-free docking using the HDOCK web server [41–43]. More stable complexes are obtained when the ligands (peptides in this study) form more chemical bonds with their receptors [44]. Accordingly, the structures were further analyzed using the Protein–Ligand Interaction Profiler (PLIP) web tool to report the amino acids as well as the chemical bonds involved in stabilizing the peptide-protein complexes [45]. The results were visualized with PyMOL software [46].

## Results

### Peptide characterization and 3D modeling

**Fig. 1:**
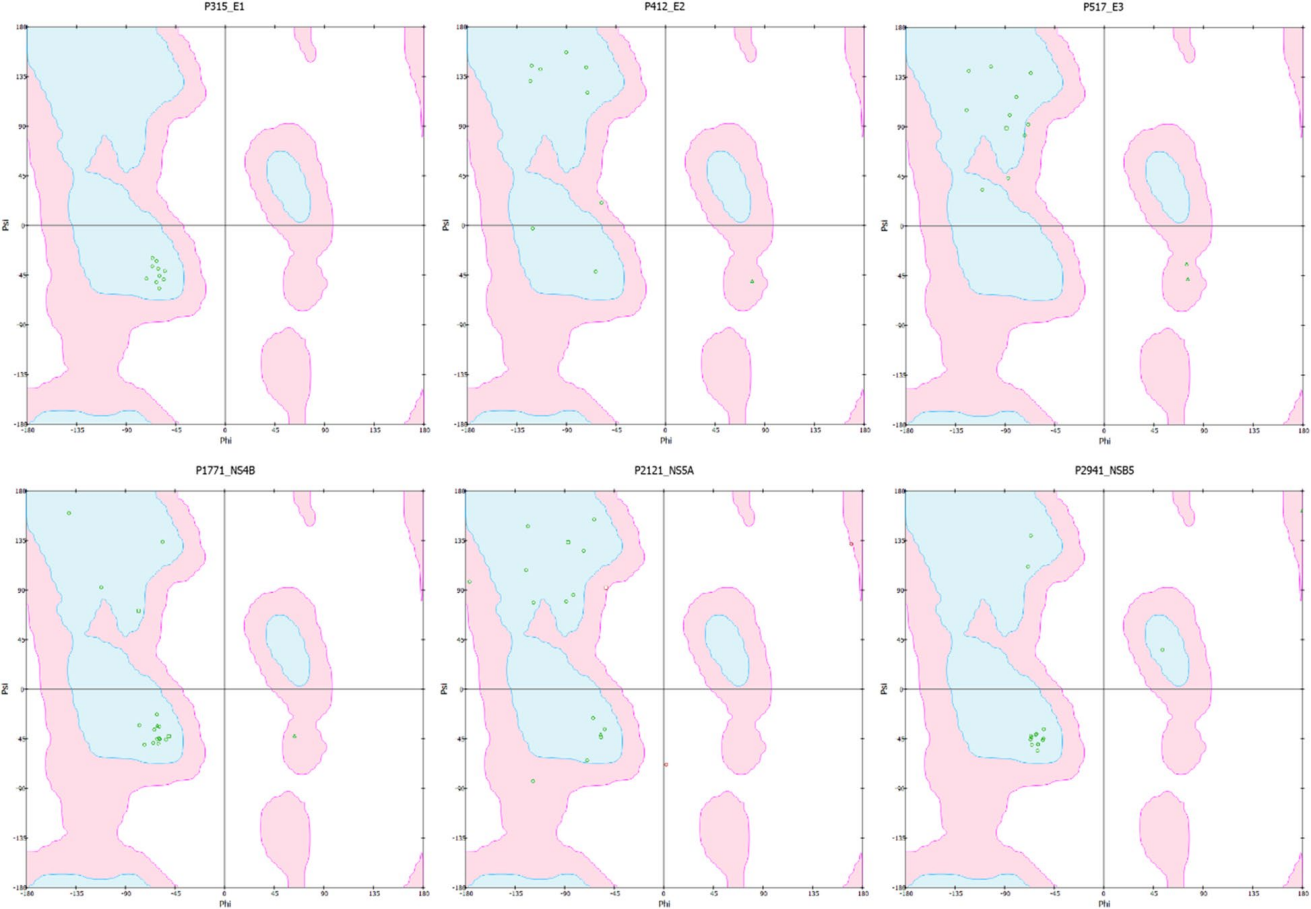
Ramachandran plots for the peptides

In general, no peptides were expected to be harmful (Table 2). Regarding the immunogenicity, only P315 and P2941 are predicted to have both strong antigenic properties and low allergenicity. Furthermore, the low sOPEP energies of P315 and P2941 indicated that they have reasonably stable structures. However, only peptide P2941 has a relatively high half-life. Despite the predicted allergenicity of peptide P1771, it has a high antigenicity and a stable 3D structure. Ramachandran plots of the peptides showed that all amino acids formed torsional angles that cause no steric clashes between atoms except in peptide P2121 in which the distances between the atoms in three residues (ASP6, PRO15, and LYS16) are shorter than the sum of their van der Waals radii which is sterically not allowed for any amino acids except glycine [47] (Fig. 1).

**Table 2:** The predicted characteristics of the peptides

Peptide	VaxiJen Score	Antigen?	Allergen?	Toxic?	Half-life (seconds)	sOPEP (kcal/mol)
P315	0.4426	Yes	No	No	841.01	-27.04
P412	0.3778	No	No	No	864.01	-13.01
P517	− 0.0809	No	Yes	No	785.11	-15.47
P1771	0.4573	Yes	Yes	No	973.61	-25.73
P2121	0.0810	No	No	No	1100.11	-23.89
P2941	0.9524	Yes	No	No	1198.51	-27.83

### Molecular docking of the peptides with their corresponding receptors

**Fig. 2:**
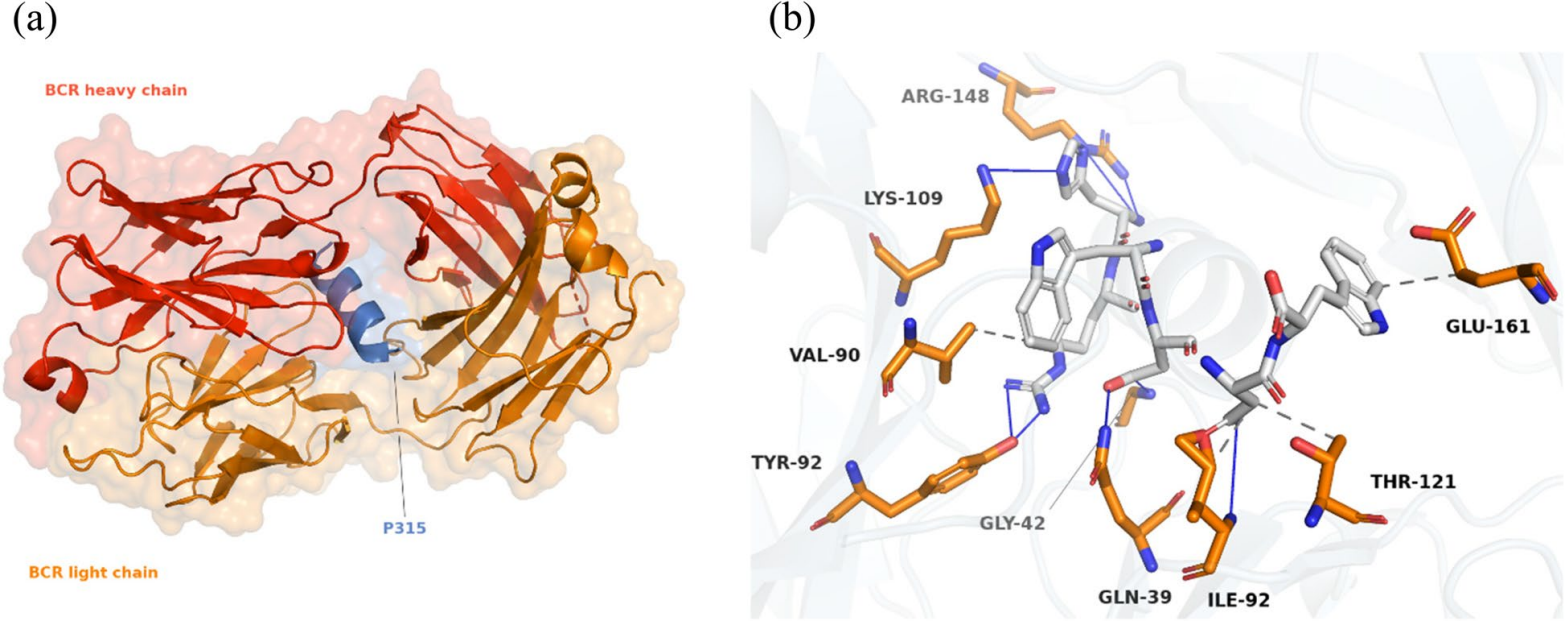
The P315-BCR docked complex shown as a whole in cartoon/surface (**a**) and in more detail in sticks view to show the chemical bonds involved in the interaction (**b**). The blue lines indicate Hydrogen bonds while the hydrophobic interactions are shown in gray dashes

**Fig. 3:**
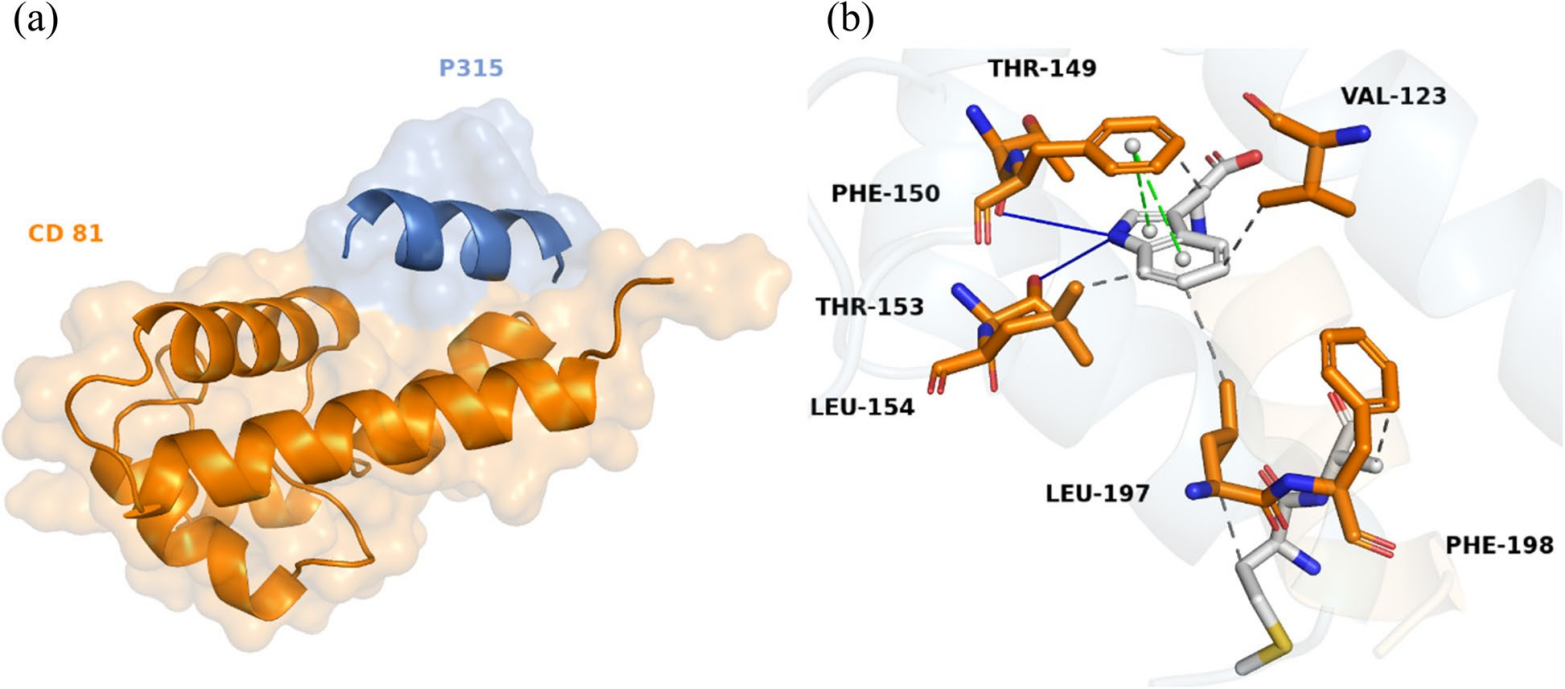
The P315-CD81 docked complex shown as a whole in cartoon/surface (**a**) and in more detail in sticks view to show the chemical bonds involved in the interaction (**b**). The blue lines indicate Hydrogen bonds while the hydrophobic interactions are shown in gray dashes. (pi interactions are shown as green dashes)

**Fig. 4:**
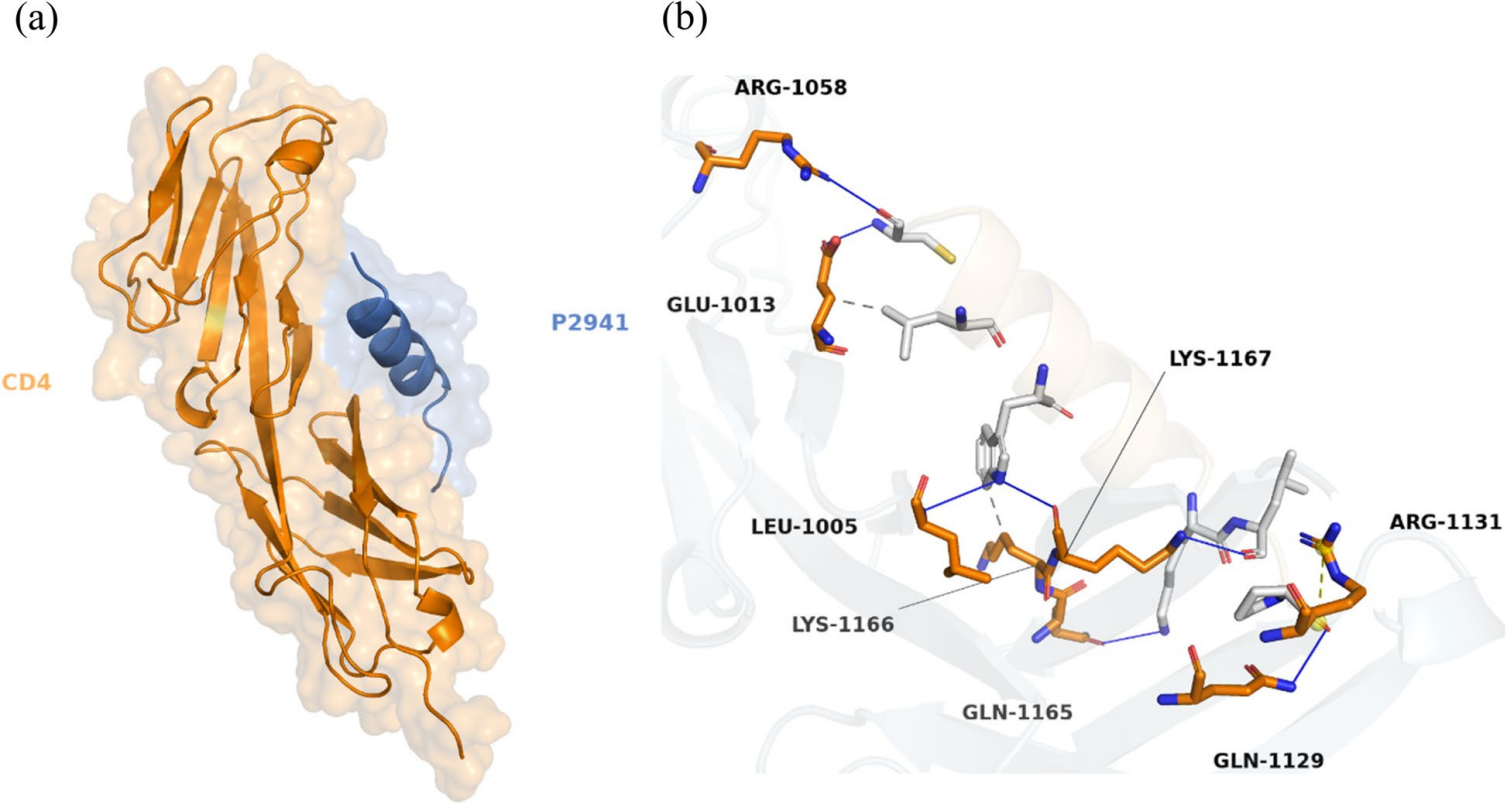
The P2941-CD4 docked complex shown as a whole in cartoon/surface (**a**) and in more detail in sticks view to show the chemical bonds involved in the interaction (**b**). The blue lines indicate Hydrogen bonds while the hydrophobic interactions are shown in gray dashes. (salt bridges are shown as yellow dashes)

**Fig. 5:**
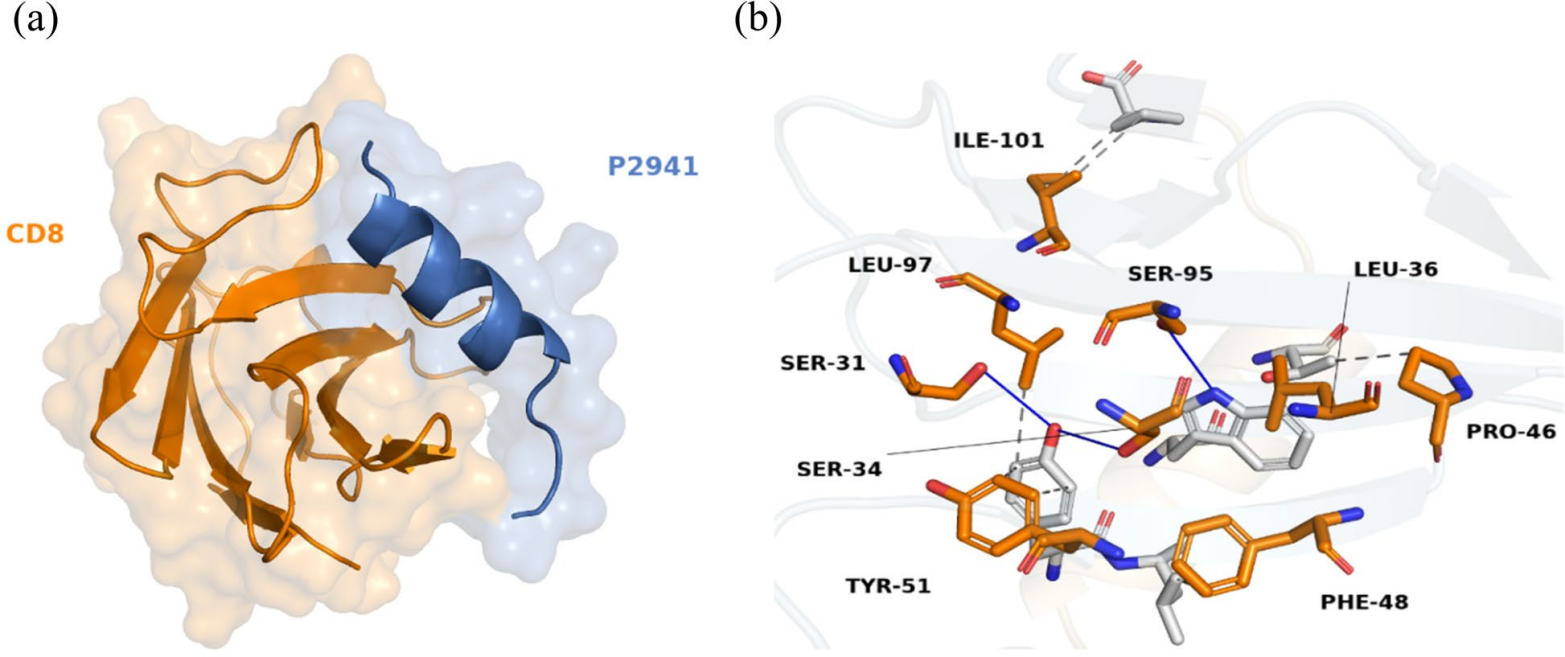
The P2941-CD8 docked complex shown as a whole in cartoon/surface (**a**) and in more detail in sticks view to show the chemical bonds involved in the interaction (**b**). The blue lines indicate Hydrogen bonds while the hydrophobic interactions are shown in gray dashes

Docking simulations revealed that peptide P315 established the most stable complexes with both targeted receptors, BCR and CD81 (Table 2). The binding of P315 with BCR, shown in Fig. 2, involved 8 hydrogen bonds and 4 hydrophobic interactions. Besides that, 6 hydrophobic and 2 hydrogen bonds contributed to the stability of the P315–CD81 complex along with 2 pi-stacked interactions, shown as green dashes in Fig. 3, formed from the ring of residue PHE-150. On the other hand, Peptide P2941 formed the most stable structures for T-cell receptors. The stable complex between P2941 and CD4 is due to the formation of 7 hydrogen bonds and a salt bridge, shown as yellow dashes, with ARG-1131 (Fig. 4). For the CD8 receptor, the interaction involved 4 hydrogen bonds and 8 hydrophobic interactions with P2941 (Fig. 5).

## Discussion

Since 2013, DAAs, a highly effective treatment has offered the best sustained virological response (SVR) ever for HCV infection [48, 49]. The SVR rates remained comparably high during the COVID pandemic (96.9% vs. pre-COVID, 98.1%), despite the widespread of intravenous drug and alcohol addiction during these challenging times [50]. Despite the achieved SVR [50, 51], the remaining greater portion of problem patients, as relapsers, and human immunodeficiency (HIV) co-infected patients are still waiting for therapy and could not be disregarded. Moreover, a scarce data is published on the long-term impact of DAAs on chronic kidney disease [52]. Up to date and according to the Centers for Disease Control and Cancer Prevention, there is no available vaccine for avoiding the occurrence of new HCV infections [53].

A considerable number of peptide vaccines are under development for HCV (using gpE2 epitope), other infectious viral diseases, and many cancers [54]. Also, wet-lab experiments generated nAb against peptides containing epitopes derived from the gpE1 and gpE2 glycoprotein [55, 56]. Recent in silico study designed a multi-epitope peptide-based vaccine against *Schistosoma mansoni* to skip the restrictions for culture maintenance of the targeted parasite. Via immunoinformatics, the Schistosoma multi-epitope vaccine was predicted as a stable, non-allergenic molecule and was hypothesized to trigger B-cell and IFN-γ-based immunity [11]. An earlier study proved the accuracy of docking to predict the binding of the stalk region of influenza hemagglutinin as an antigen with two different antibodies [57].

Herein, a set of computational methods was employed to demonstrate the efficacy of the individual peptides used in the six HCVP6-MAP cocktails to achieve the desired immunogenicity against HCV infection. As toxicity is one of the major parameters in selecting the ideal vaccine [58], Our results revealed that all tested peptides didn’t have any toxicity. Moreover, for humoral and cellular responses, P315 and P2941 are predicted to have robust antigenic properties, lowest allergenicity, and minimal sOPEP energies. All these parameters conferred the stability of the tested peptides. Regarding the stability in blood, the P2941, in addition and relative to other peptides, has the longest half-life time. While the peptide P1771 had antigenic power, considerable half-life time, and low sOPEP energy with a stable 3D structure.

By using HDOCK, a server for protein–protein docking strategy, several combinations of different receptors with the six studied peptides were explored to estimate the docking performance in all potential scenarios. Regarding B cell receptors, the docking results showed that P315 formed the highest hydrophobic bonds with the CD81 receptor. As with many tetraspanins, CD81 is the mediator for HCV entry via binding to cholesterol in a cavity formed by its transmembrane domains. The latter receptor participates not only in HCV cell-surface assembly but also in *Plasmodium* sporozoites, HIV, and influenza A virus. Upon mutating the hydrogen bonds between cholesterol and CD81, HCV entry showed a 50% reduction [59]. Furthermore, the strength of hydrogen bonds submerged in the protein interior is reported to be as high as 7 kJ/mol per bond [60]. P412 peptide showed a stable complex with the BCR (7 hydrogen bonds) and modest energy (− 230), while P315 conferred the lowest energy (− 233) with 8 hydrogen bonds for the same receptor. Moreover, the latter peptide contains amino acid residues (as L413, G418, W420, G523, P525, Y527, W529, and G530) involved in CD81 blockade by improving nAb epitope exposure via inhibiting E2-CD81 receptor interactions [55, 61]. Conversely, with CD81 receptors, P517 exhibited the minimum number of hydrogen bonds and has minimal hydrophobicity (Table 3).

**Table 3:** Docking scores of the peptides with the receptors and the number of formed chemical bonds

Response	Receptor	Peptide	Docking score	Hydrogen bonds	Hydrophobic interactions
**B-cell**	BCR	P315	− 233.4	8	4
		P412	− 230.9	7	2
		P517	− 219.8	5	3
	CD81	P315	− 235.0	2	6
		P412	− 219.9	2	5
		P517	− 218.4	1	2
**T-cell**	CD4	P1771	− 188.2	3	5
		P2121	− 213.5	4	4
		P2941	− 228.1	7	2
	CD8	P1771	− 229.9	2	5
		P2121	− 268.1	4	5
		P2941	− 274.9	4	8

The current in silico study employed a set of computational tools to evaluate and characterize the antigenicity and receptor-binding for each peptide individually to determine the most effective structural and non-structural peptides in terms of triggering the humoral and cellular responses. the current in silico study employed a set of computational tools to evaluate and characterize the antigenicity and receptor-binding for each peptide individually to determine the most effective structural and non-structural peptides in terms of triggering the humoral and cellular responses. the current in silico study employed a set of computational tools to evaluate and characterize the antigenicity and receptor-binding for each peptide individually to determine the most effective structural and non-structural peptides in terms of triggering the humoral and cellular responses. the current in silico study employed a set of computational tools to evaluate and characterize the antigenicity and receptor-binding for each peptide individually to determine the most effective structural and non-structural peptides in terms of triggering the humoral and cellular responses. the current in silico study employed a set of computational tools to evaluate and characterize the antigenicity and receptor-binding for each peptide individually to determine the most effective structural and non-structural peptides in terms of triggering the humoral and cellular responses. the current in silico study employed a set of computational tools to evaluate and characterize the antigenicity and receptor-binding for each peptide individually to determine the most effective structural and non-structural peptides in terms of triggering the humoral and cellular responses. the current in silico study employed a set of computational tools to evaluate and characterize the antigenicity and receptor-binding for each peptide individually to determine the most effective structural and non-structural peptides in terms of triggering the humoral and cellular responses.

## Conclusion

Taken together, the strongest antigenicity and the lowest allergenicity were shown by P315. In turn, P315 (derived from gpE1) was shown to strongly bind to the BCR and CD81 receptors that will elicit B cell function. In our results, P2941 (derived from NS5B), that are known to stimulate a CD4 + T cell response specific to HCV was shown to strongly bind to both CD4 and CD8 receptors. No doubt that the 3D designs of human immunogenic targets make it possible to find new drugs and vaccines, while their crystallization and purification continue to be rate-determining processes. But further research on peptide vaccines could lead to the production of safe, effective HCV vaccines for use in clinical trials.

## Data Availability

All analyzed data are available from the corresponding author on reasonable request.

## Abbreviations

HCV: Hepatitis C Virus
HCC: Hepatocellular carcinoma
DAAs: Direct-acting antiviral therapy
BCR: B-cell receptor
CDC: Centers for Disease Control and Cancer Prevention
E1 and E2: Envelope proteins
PLIP: Protein-Ligand Interaction Profiler
HIV: Human immunodeficiency
SVR: Sustained virological response
